# Causal relationship between iron deficiency anemia and asthma: a Mendelian randomization study

**DOI:** 10.3389/fped.2024.1362156

**Published:** 2024-05-23

**Authors:** Mengchun Li, Zhengdong Chen, Xin Yang, Wanwei Li

**Affiliations:** Department of Pediatrics, Daping Hospital, Army Medical University, Chongqing, China

**Keywords:** iron deficiency anemia, asthma, Mendelian randomization, risk, prevention

## Abstract

**Background:**

Observational studies have suggested an association between iron deficiency anemia (IDA) and asthma, which may affect the occurrence of asthma. However, whether IDA is a new management goal for asthma remains to be determined.

**Objective:**

We conducted a two-sample Mendelian randomization(MR)analysis to assess the association between IDA and asthma.

**Methods:**

We performed a two-sample MR study to assess a causal relationship between IDA (ncase = 12,434, ncontrol = 59,827) and asthma (ncase = 20,629, ncontrol = 135,449). Inverse variance weighted (IVW) was used as the primary method for the analyses. Furthermore, we used weighted medians and MR-Egger to enhance robustness. Data linking genetic variation to IDA and asthma were combined to assess the impact of IDA on asthma risk.

**Results:**

There are five single nucleotide polymorphisms (SNPs) were used as genetic tool variables for exposure factors. Genetically determined IDA was significantly associated with an increased risk of asthma (OR = 1.37, 95% CI: 1.09–1.72, *p* = 0.007). There was little heterogeneity in the MR studies and no evidence of level pleiotropy was found.

**Conclusions:**

In our MR study, our findings emphasize that IDA may be associated with a high risk of asthma, indicating a potential role for IDA in the development of asthma. Future research needs to elucidate its potential mechanisms to pave the way for the prevention and treatment of asthma.

## 1 Introduction

Asthma is one of the most common chronic diseases in the world, with a mortality rate of (1.6–36.7)/100,000, making it a major health problem worldwide (1). It is characterized by varying degrees of airflow obstruction, which can cause dyspnea and wheezing, and asthma management consists of assessment of asthma control and risk factors and the appropriately adjusted medication cycle (2). Globally, asthma ranks 16th among the leading causes of years lived with disability and 28th among the leading causes of disease burden, as measured by disability-adjusted life years (3). According to the 2019 Global Burden of Disease (GBD) Asthma Study, the global disability-adjusted life years (DALYs) of asthma were 9.9% and 8.8% respectively, and approximately 81 million children worldwide suffer from asthma (4). By 2025, an additional 100 million people may be affected (5). Asthma represents a significant disease burden globally, driving the need for prevention and treatment. Although the etiology of asthma remains largely unknown, it suggests that genetic background, environmental factors, and their interactions play a crucial role in the development of asthma.

Asthma is a complex inflammatory disease of the airways with multiple pathophysiological features. Type 2 (T2) inflammation is an important immune response in the pathobiology of asthma, leading to the classification of asthma into T2-high and T2-low (6), eosinophils are the most important inflammatory cells in T2 high asthma (7), and they are key effector cells that contribute to the pathogenesis of asthma by inducing type 2 inflammation, the primary asthma trigger (8, 9). Many studies have shown a correlation between elevated blood eosinophil levels and acute asthma exacerbations and asthma severity (10). There are many documents showing that the probability of anemia in patients with asthma is significantly increased (11–13). Iron is one of the important trace elements that is essential for many biological processes, including the regulation of enzyme activity, oxygen transport, and immune function (14, 15), anemia affects a decrease in the strength of the respiratory muscles, including the diaphragm, and reduces lung function (16), which may be a mechanism affecting asthma. Dietary iron supplementation has been reported to reduce airway eosinophilia in animal models to reduce the severity of allergic asthma (17). But research results on the relationship between IDA and asthma are inconclusive.

Anemia is a common disease that may have a significant impact on health and represents a heavy global burden. Developing countries account for 89% of all anemia-related disabilities (18), and IDA remains the main cause of anemia. Genotype is defined at the time of pregnancy and is often not associated with traditional confounders in observational studies (19).

MR is a data analysis technique for assessing the inference of etiology in epidemiological studies. It uses genetic variation as an instrumental variable (IV) in non-experimental data to estimate the causal relationship between the exposure factor of interest and outcome of concern (20, 21), where exposure factor refers to a putative causal risk factor, also known as intermediate phenotypes, which can be biological, anthropometric, or any risk factor that may influence resolution. Diseases are generally listed as an outcomes, but are not limited to a specific disease. Non-experimental data cover all observational studies, including cross-sectional studies, longitudinal series, cohort studies, and case-control studies. MR involves the use of genetic markers associated with exposure SNPs to test the causal relationship between exposure and outcome (22). This design is less likely to be confounded or influenced by reverse causality due to the random distribution of alleles during gamete formation. MR can effectively reduce the impact of confounding factors that may affect exposure and outcome phenotypes. With the booming development of publicly available large sample size genome-wide association studies (GWAS) data, higher statistical power can be obtained more effectively. Previously, MR analysis has been used to explore the causal relationship between anemia and many diseases, such as cardiovascular disease (23), chronic obstructive pulmonary disease (24), and depression (25). But it has not yet been applied to study its impact on asthma. Therefore, we performed a two-sample MR analysis to test whether genetically determined IDA is causally related to the development of asthma.

## 2 Materials and methods

### 2.1 Study design and instrumental variants selection

The MR study was based on a large-scale GWAS summary data set. In all of these corresponding original studies, all participants gave informed consent and no additional ethical approval was required because we used only summary-level statistics. The primary analysis was based on IDA (*n* = 72,261) from the IEU consortium on asthma (*n* = 156,078) from the IEU consortium. Sensitivity analysis includes Cochran's *Q* test, leave-one-out analysis, funnel plots, and MR-Egger intercept analysis.

**Figure 1:**
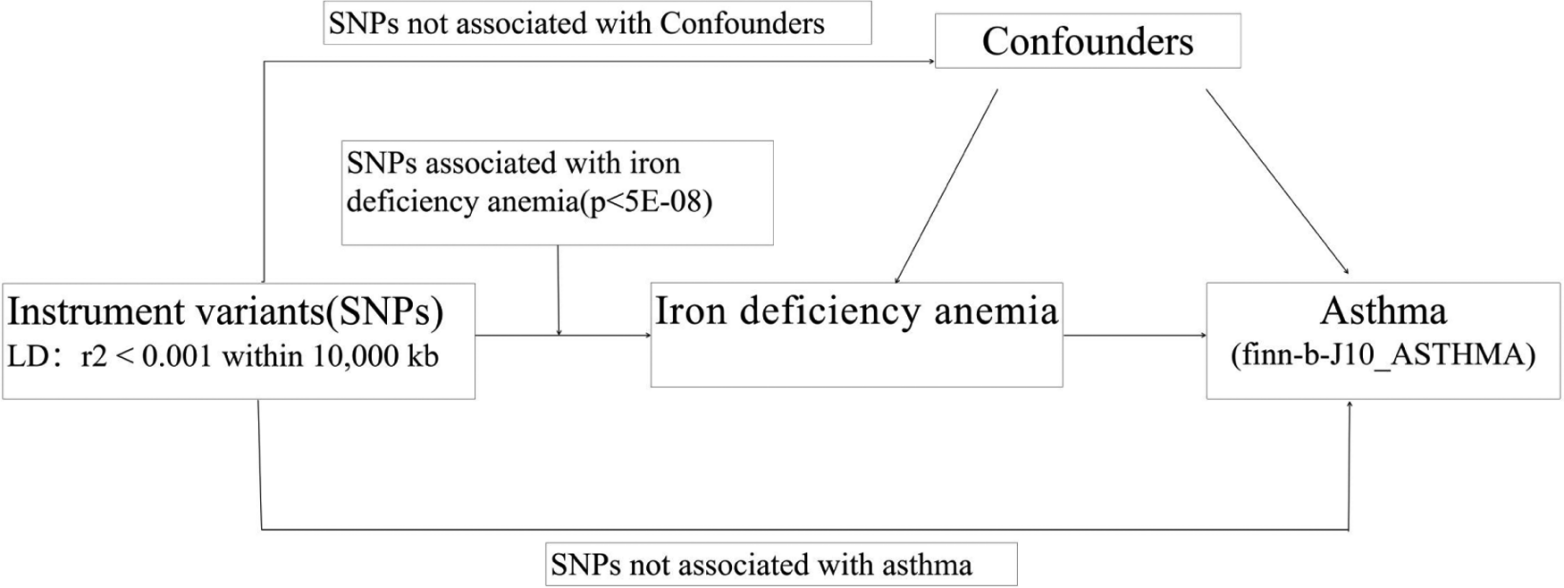
The flowchart in this two-sample Mendelian randomization study. SNPs:single nucleotide polymorphisms.

As presented in Figure 1, the two-sample MR study must meet three principal assumptions. According to assumption 1, genetic instrument variants are closely related to IDA (exposure factor). Assumption 2 is that SNPs are not associated with any other disease other than exposure and outcome. Assumption 3 was that the risk of outcome (asthma) was strongly influenced by IDA genetic instrument variants through the exposure factor IDA but not through other pathways (26). As previously described, the second and third assumptions are collectively known as independence from pleiotropy. In addition, we also searched in the phenoscanner performed IVW analysis to evaluate whether there is a causal effect of IDA on potential risk factors for asthma, including viral infection, body mass index (BMI), and obesity.

### 2.2 Genetic variants associated with IDA

The primary genetic tool comes from the IDA's GWAS dataset recently collected by the IEU consortium, which includes 72,261 individuals of European ancestry. SNPs closely associated with IDA (*p* < 5 × 10^−8^) were selected as IVs. To further obtain independent SNPs, we then pruned these instruments within a window size of 10,000 kb to mitigate linkage disequilibrium (LD) at a threshold of *r*^2^ < 0.001. For SNPs, the *F* statistic was used to evaluate instrumental variables and exposure. The strength of the correlation between factors, only when the *F* statistic is >10, it indicates good stability.

### 2.3 Asthma genome-wide association studies datasets

Anemia statistics included a total of 12,434 anemia cases and 59,827 controls of European ancestry (OpenGWAS: finn-b-D3_ANAEMIA) from the FinnGen Consortium. Asthma statistics included a total of 20,629 asthma cases and 135,449 controls of European ancestry (OpenGWAS: finn-b-J10_ASTHMA) from the FinnGen Consortium. The inclusion and exclusion criteria and the detailed baseline characteristics of studied patients were derived from the FinnGen Consortium (27) (https://r5.risteys.finngen.fi/phenocode/D3_ANAEMIA#, https://r5.risteys.finngen.fi /phenocode/J10_ASTHMA#).

### 2.4 Mendelian randomization analysis

#### 2.4.1 Pleiotropy, heterogeneity, and power analysis

The exposure SNPs were extracted from the full GWAS data of asthma. Coordinated processing was then performed to ensure that the exposed effector alleles were aligned with the resulting SNPs, excluding SNPs with incompatible alleles or palindromes with moderate effector allele frequencies. More precisely, there are four steps. First, we aggregate SNPs to obtain independent genetic instrumental variables. Second, for missing SNPs, the proxy SNPs can be found. Third, we excluded SNPs that were significantly correlated with the outcome. Fourth, ambiguous and palindromic SNPs were discarded. Then, the MR was analyzed. Specifically, IVW estimates were used as the primary MR effect estimates, reported as odds ratios (ORs) and 95% confidence intervals (95% CIs), where a fixed effect model was used (20). When directional pleiotropy is absent, the IVW method can deliver a relatively stable and precise causal evaluation by using a meta-analytic approach to combine Wald estimates for each IV. We also estimated causal effects using two other methods: weighted median and MR-Egger regression methods. The MR-Egger method can provide a relatively robust estimate without the influence of the validity of IVs, and an adjusted result by existing horizontal pleiotropy via the regression slope and intercept. These three methods are considered to be the most scientific and commonly used methods to provide robust analysis of the results of MR surveys (28). If weighted median method is to be used, at least 50% of SNPs must meet the premise that they are valid instrumental variables. The adaptability of MR-Egger can detect some violations of the standard instrumental variable assumptions and provide an effect estimate that is not constrained by these violations (28).

Sensitivity analysis is an essential method for assessing potential bias in mendelian randomization studies. It includes the following two considerations, heterogeneity testing and pleiotropy testing. The leave-one-out sensitivity test was used to judge the stability of the MR results by excluding IVs one by one. The heterogeneity of the IVW assay was determined by the Cochran's *Q* test. The MR pleiotropy residual sum and outlier test (MR-PRESSO) test with default parameters was used to identify abnormal values for horizontal polymorphisms, and the MR-Egger intercept test was used to assess the presence of potential horizontal pluripotency driven MR results (the presence of *p* < 0.05 intercept for horizontal pluripotency) (29).

MR analysis was performed with R software (version 4.3.0), the TwoSample MR software package (version 0.5.6), and the RadialMR software package (version 1.0). IVW providing a robust causal assessment in the absence of directional pleiotropy, is used as the main method for calculating causal estimates between IDA and asthma. The main assumptions and flowchart of this study are shown in Figure 1.

## 3 Results

### 3.1 Primary analyses

**Figure 2:**
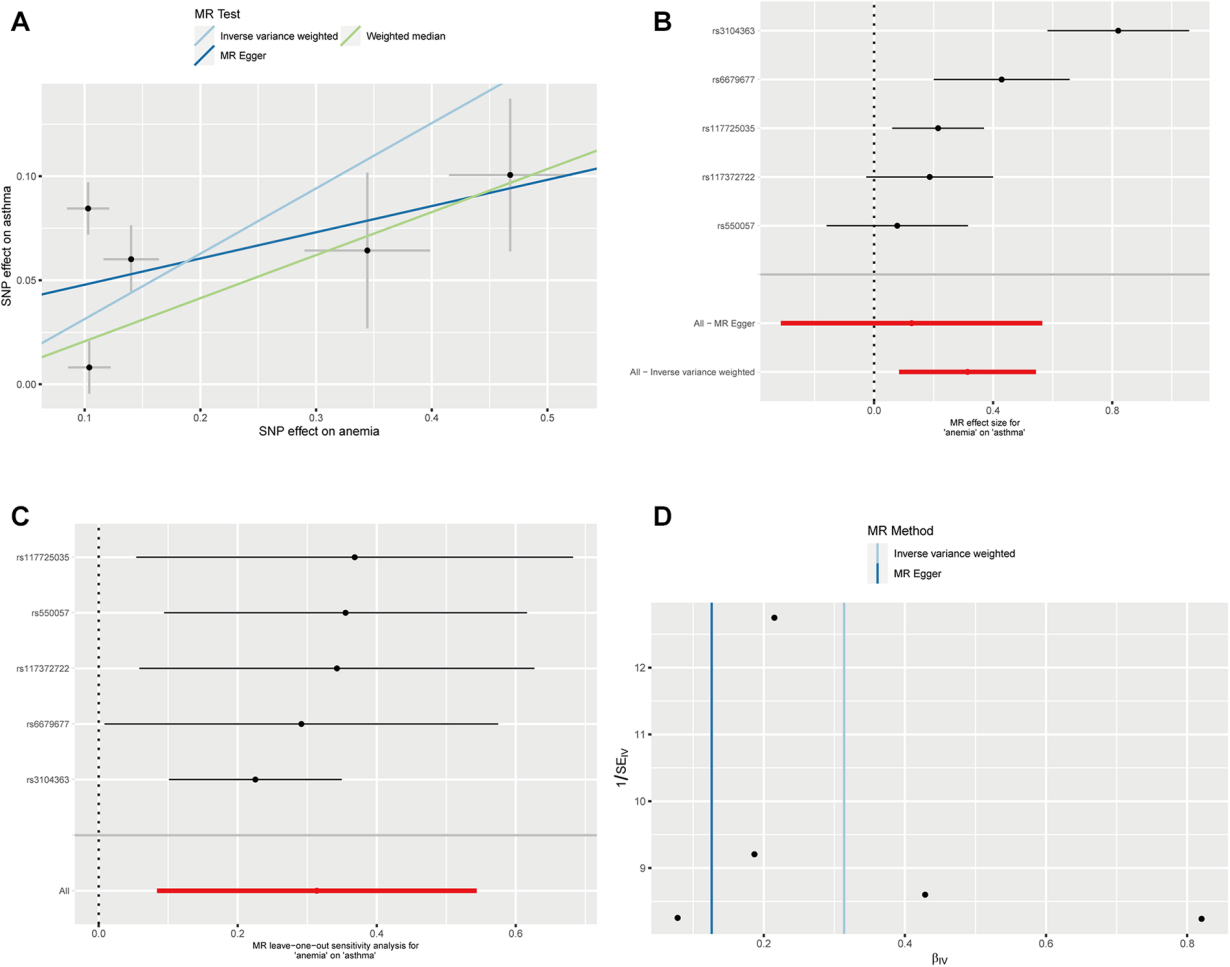
Mendelian randomization estimates from IDA on asthma. (**A**) Scatter plot showing the causality of IDA on asthma by MR-PRESSO and MR Radial; (**B**) Forest plots of the IVW estimates; (**C**) susceptivity analysis; (**D**) Funnel plots.

We successfully extracted five corresponding IDA-associated genetic variants from the asthma GWAS data set. We assessed the causal relationship between IDA and asthma using IVW, MR-Egger regression, and weighted median are summarized in Figure 2. The IVW results showed strong evidence for a causal relationship between IDA and the risk of asthma (OR = 1.37, 95% CI: 1.09–1.72, *p* = 0.007). At the same time, similar risk estimates were obtained using and weighted median (OR = 1.23, 95% CI = 1.08–1.40, *p* = 0.002). The consistency of the three MR models enhanced the reliability of IDA as a proxy for risk of asthma. However, there was no evidence of a significant intercept (intercept = 0.0352, *p* = 0.397), indicating no observed pleiotropy. The results of the multi-validation and heterogeneity analyses are summarized in Table 1.

**Table 1:** Pleiotropy and heterogeneity test in MR analyses.

Pleiotropy test			Heterogeneity test					
MR_egger			MR_egger			IVW		
Intercept	SE	pval	*Q*	*Q*_df	*Q*_pval	*Q*	*Q*_df	*Q*_pval
0.0352	0.0358	0.3972	18.998	3	<0.001	25.145	4	<0.001

### 3.2 Summary of the Mendelian randomization analysis

Causality estimates (OR and 95% CI) of IVW with *p* values are presented in the forest plot. The results of the MR-PRESSO global study for *p*-values, MR-Egger intercepts and *p*-values, IVW heterogeneity, and MR Egger tests are shown in Table 1. Causality estimates were calculated using weighted medians and Mr-Egger's method.

### 3.3 Causal effects of IDA on asthma

**Figure 3:**
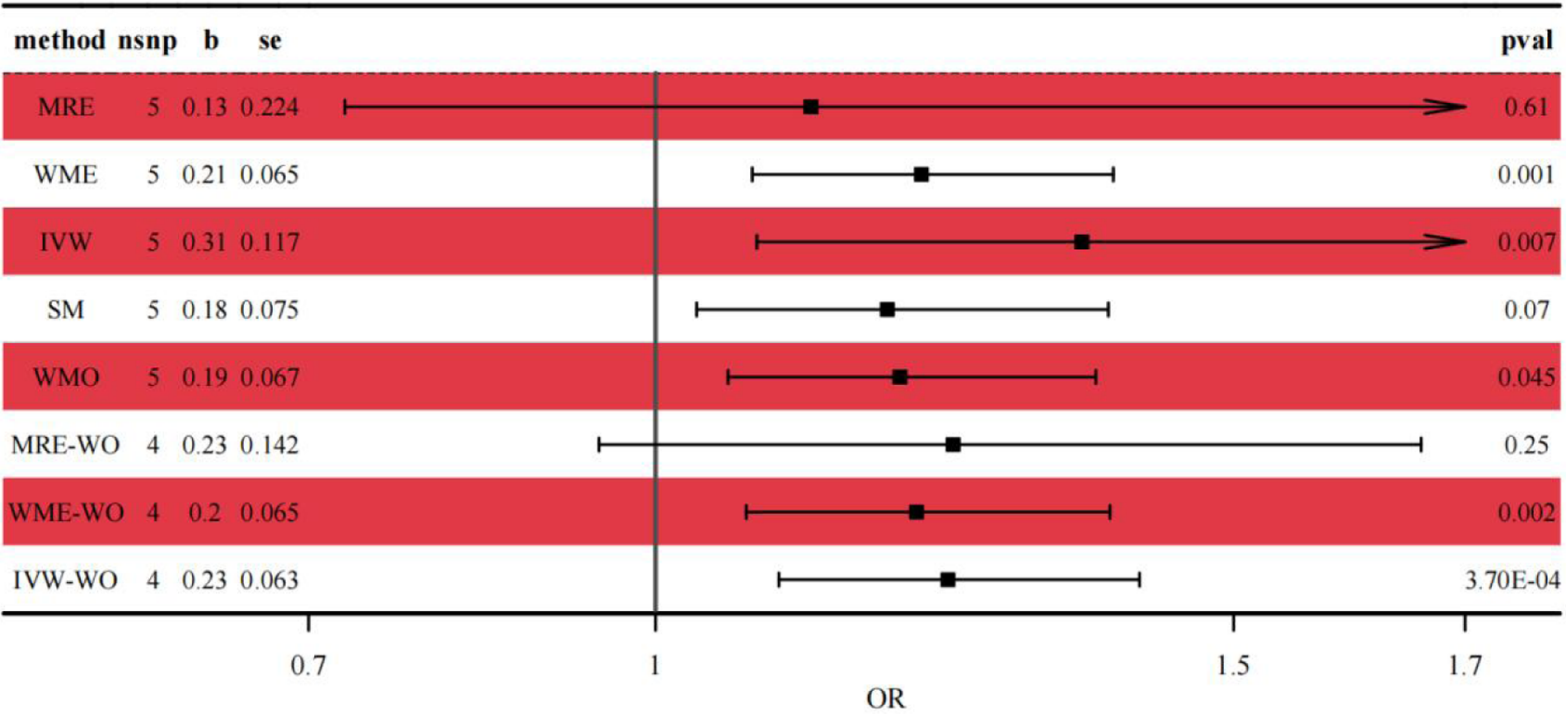
Forest plot of the casual relationship between IDA and asthma. MRE, MR-egger; WME, weighted median; IVW, inverse variance weighted; SM, simple mode; WMO, weighted mode; WO, without outliers.

Genetic prediction of IDA was significantly associated with increased risk of asthma using the IVW method (OR = 1.37, 95% CI: 1.09–1.72, *p* = 0.007) and similar results were found using weighted medians (OR = 1.230, 95% CI: 1.087–1.391). No significant directional level of pleiotropy was found on the MR-egger test (*p* = 0.3972). Furthermore, heterogeneity was suggested in the MR-egger (Cochran *Q* = 18.998, *p* < 0.001) and IVW (Cochran *Q* = 25.145, *p* < 0.001) regression tests in Figure 3.

### 3.4 Power analysis

In our study, the F statistic ranged from 417–822, with values greater than 10, indicating a strong potential for predicting IDA levels.

## 4 Discussion

While various studies have been conducted to explore the association between IDA and asthma, this study is the first MR analysis to assess the causal relationship between IDA and susceptibility to asthma. Using the IVW approach, our results suggest that genetically predicted IDA is causally linked to an increased risk of asthma, suggesting that IDA may be a potential factor in the development of asthma.

Our findings are consistent with a large number of observational studies from different countries that report on the relationship between anemia and asthma (13, 30–32). However, some observational studies have reported that asthma increases the risk of anemia (11, 30, 33), but we believe that the causal relationship we found may be more reliable than the results of observational studies because MR analysis is less susceptible to confounding or effects of reverse causation. Our analysis of different analytical methods provides strong support for the assessment of IDA as a cause of asthma.

Asthma is a common allergic disease caused by immune system disorders, which is characterized by chronic inflammation, airway hyperresponsiveness and periodic airflow obstruction. Anemia and asthma are both high-prevalence diseases in children (9), which have a negative impact on children's growth, comorbidities, and health-related quality of life (12). IDA can cause many deleterious effects on humans, such as pregnancy, fetal development, and child growth (34), and is also associated with the risk of many diseases (35, 36). For example, previous studies have shown that IDA is associated with an increased risk of autism, Parkinson's disease, and cancer (29, 37–39). Anemia or low iron levels during pregnancy are associated with an increased risk of asthma in the offspring (40–42). In US women, higher iron stores are negatively associated with asthma (43).

Numerous studies have shown that IDA is a common phenomenon in asthma (11–13). Previous studies have shown that immune dysfunction also plays a crucial role in the development and of asthma (44). As a chronic airway disease, asthma has long been considered predominantly an inflammatory disease. Iron is an important trace element that plays a key role in various biological processes such as the regulation of enzyme activity, oxygen transport, and immune functions, and may affect the development of asthma. Iron deficiency can adversely affect the immune response, and low hemoglobin can impair tissue oxygenation and serve as an independent risk factor for LRTI in children (45).

Immune activation or inflammation in patients with asthma is associated with iron deficiency. Macrophages are present in all tissues and their function is to support and restore tissue homeostasis, in addition to serving as a sensor for iron requirements in tissues and for the supply of iron as an essential trace element (46). These iron-treatment characteristics determine anti- and inflammatory states (47). Several studies have found an association between iron deficiency and atopic disease. Shaheen's study reported that the direct link between asthma and iron deficiency is obvious (42). In animal models, iron administration suppresses airway hyperresponsiveness and eosinophilia, suggesting that higher iron status may be protective in asthma (48). A low-iron diet causes overt asthma in a mouse model of allergic asthma, which is mediated by increased mast cell reactivity in a low-iron environment. Clinical and experimental evidence suggests that alterations in the levels of systemic and pulmonary iron and/or iron regulatory molecules are associated with lung inflammation in many diseases, including asthma (49). Caffarelli's research showed that obese children had higher rates of asthma, higher hepcidin levels and lower iron levels. Animal experiments have shown that iron is related to fetal airway development (50). Ramakrishnan et al., through a prospective (cohort) study, found that anemic children were 5.75 times more likely to have an asthma attack than non-anemic children (16). Hemoglobin facilitates the transport of oxygen and carbon dioxide. It carries and inactivates nitric oxide (NO) and also acts as a buffer. Hemoglobin in the blood is primarily responsible for stabilizing oxygen tension in tissues, and qualitative and/or quantitative reductions in Hb may adversely affect normal function.

In epidemiological studies, the possible impact of IDA on the development of asthma has been extensively investigated, but few of them provide strong evidence of a causal relationship. Our findings provide evidence that IDA is associated with asthma, This may mean that active iron supplementation in children with IDA may reduce the occurrence of asthma, and improving anemia may be effective for asthma patients in clinical practice. However, the diagnosis of anemia in asthma patients is easily ignored in clinical practice. Therefore, we recommend active clinical monitoring of anemia in children with asthma.

## 5 Strength and limitation

There are several clear advantages in our study. No pleiotropy were found in the MRPRESSO test and MREgger Intercept test, suggesting the good reliability of our results. Also, after deleting outliers, the result were still significant. Furthermore, the causal estimates obtained from all methods are positively related, providing a robust result to our research.

Because of several limitations of this study, our findings should be interpreted with caution. MR performed sensitivity analyzes to prevent selection bias against genetic variants with pleiotropic effects on the results. First, all the genetic data used in this study were from Europeans, and it remains to be further confirmed whether the causal relationship between IDA and asthma observed in this study is also common in other ethnic groups. Second, the data are only from the FinnGen Consortium and are not all GWAS data for children, so there are limitations regarding the impact of IDA on the increased risk of asthma in children. Third, we cannot rule out that our analyzes were confounded by mediation effects. These should be considered in future work.

## 6 Conclusions

The results of our MR study suggest that genetically predicted IDA may be causally associated with an increased risk of asthma. Our findings alert clinicians that more attention should be paid to iron status in children with a family history of asthma. Elucidating the underlying mechanisms by which IDA contributes to asthma susceptibility is critical to guide the prevention and treatment of asthma, and further research should identify the molecular and pathways through which IDA can initiate asthma.

## Data Availability

The original contributions presented in the study are included in the article/Supplementary Material, further inquiries can be directed to the corresponding authors.

## References

[1] Asher MI Montefort S Björkstén B Lai CK Strachan DP Weiland SK Worldwide time trends in the prevalence of symptoms of asthma, allergic rhinoconjunctivitis, and eczema in childhood: ISAAC phases one and three repeat multicountry cross-sectional surveys. Lancet. (2006) 368(9537):733–43. 10.1016/S0140-6736(06)69283-0 16935684

[2] Porsbjerg C Melén E Lehtimäki L Shaw D. Asthma. Lancet. (2023) 401(10379):858–73. 10.1016/S0140-6736(22)02125-0 36682372

[3] Dharmage SC Perret JL Custovic A. Epidemiology of asthma in children and adults. Front Pediatr. (2019) 7:246. 10.3389/fped.2019.00246 31275909 PMC6591438

[4] Safiri S Carson-Chahhoud K Karamzad N Sullman MJM Nejadghaderi SA Taghizadieh A Prevalence, deaths, and disability-adjusted life-years due to asthma and its attributable risk factors in 204 countries and territories, 1990–2019. Chest. (2022) 161(2):318–29. 10.1016/j.chest.2021.09.042 34699773

[5] To T Stanojevic S Moores G Gershon AS Bateman ED Cruz AA Global asthma prevalence in adults: findings from the cross-sectional world health survey. BMC Public Health. (2012) 12:204. 10.1186/1471-2458-12-204 22429515 PMC3353191

[6] Kaur R Chupp G. Phenotypes and endotypes of adult asthma: moving toward precision medicine. J Allergy Clin Immunol. (2019) 144(1):1–12. 10.1016/j.jaci.2019.05.031 31277742

[7] Schleich FN Chevremont A Paulus V Henket M Manise M Seidel L Importance of concomitant local and systemic eosinophilia in uncontrolled asthma. Eur Respir J. (2014) 44(1):97–108. 10.1183/09031936.00201813 24525441

[8] Woodruff PG Modrek B Choy DF Jia G Abbas AR Ellwanger A T-helper type 2-driven inflammation defines major subphenotypes of asthma. Am J Respir Crit Care Med. (2009) 180(5):388–95. 10.1164/rccm.200903-0392OC 19483109 PMC2742757

[9] Licari A Manti S Castagnoli R Marseglia A Foiadelli T Brambilla I Immunomodulation in pediatric asthma. Front Pediatr. (2019) 7:289. 10.3389/fped.2019.00289 31355170 PMC6640202

[10] Tran TN Khatry DB Ke X Ward CK Gossage D. High blood eosinophil count is associated with more frequent asthma attacks in asthma patients. Ann Allergy Asthma Immunol. (2014) 113(1):19–24. 10.1016/j.anai.2014.04.011 24846699

[11] Rhew K Choi J Kim K Choi KH Lee SH Park HW. Increased risk of anemia in patients with asthma. Clin Epidemiol. (2023) 15:31–8. 10.2147/CLEP.S394717 36636733 PMC9830059

[12] Chang JE Lee HM Kim J Rhew K. Prevalence of anemia in pediatric patients according to asthma control: propensity score analysis. J Asthma Allergy. (2021) 14:743–51. 10.2147/JAA.S318641 34234469 PMC8254559

[13] Rhew K Oh JM. Association between atopic disease and anemia in pediatrics: a cross-sectional study. BMC Pediatr. (2019) 19(1):455. 10.1186/s12887-019-1836-5 31760939 PMC6876088

[14] Wen J Wang C Xia J Giri M Guo S. Relationship between serum iron and blood eosinophil counts in asthmatic adults: data from NHANES 2011–2018. Front Immunol. (2023) 14:1201160. 10.3389/fimmu.2023.1201160 37731511 PMC10507334

[15] Thibault H Galan P Selz F Preziosi P Olivier C Badoual J The immune response in iron-deficient young children: effect of iron supplementation on cell-mediated immunity. Eur J Pediatr. (1993) 152(2):120–4. 10.1007/BF02072487 8444218

[16] Ramakrishnan K Borade A. Anemia as a risk factor for childhood asthma. Lung India. (2010) 27(2):51–3. 10.4103/0970-2113.63605 20616934 PMC2893424

[17] Maazi H Shirinbak S Bloksma N Nawijn MC van Oosterhout AJ. Iron administration reduces airway hyperreactivity and eosinophilia in a mouse model of allergic asthma. Clin Exp Immunol. (2011) 166(1):80–6. 10.1111/j.1365-2249.2011.04448.x 21910724 PMC3193922

[18] Kassebaum NJ. The global burden of anemia. Hematol Oncol Clin North Am. (2016) 30(2):247–308. 10.1016/j.hoc.2015.11.002 27040955

[19] Smith GD Ebrahim S. "Mendelian randomization": can genetic epidemiology contribute to understanding environmental determinants of disease. Int J Epidemiol. (2003) 32(1):1–22. 10.1093/ije/dyg070 12689998

[20] Bowden J Holmes MV. Meta-analysis and Mendelian randomization: a review. Res Synth Methods. (2019) 10(4):486–96. 10.1002/jrsm.1346 30861319 PMC6973275

[21] Birney E. Mendelian randomization. Cold Spring Harb Perspect Med. (2022) 12(4):a041302. 10.1101/cshperspect.a041302 34872952 PMC9121891

[22] Emdin CA Khera AV Kathiresan S. Mendelian randomization. JAMA. (2017) 318(19):1925–6. 10.1001/jama.2017.17219 29164242

[23] Gan T Hu J Liu W Li C Xu Q Wang Y Causal association between anemia and cardiovascular disease: a 2-sample bidirectional Mendelian randomization study. J Am Heart Assoc. (2023) 12(12):e029689. 10.1161/JAHA.123.029689 37301769 PMC10356041

[24] Wei Y Sun L Liu C Li L. Causal association between iron deficiency anemia and chronic obstructive pulmonary disease: a bidirectional two-sample Mendelian randomization study. Heart Lung. (2023) 58:217–22. 10.1016/j.hrtlng.2023.01.003 36623443

[25] Wang Y Guo D Sui C Qu Z He G Meng H Association between anemia and depression: results from NHANES 2005–2018 and Mendelian randomization analyses. Ann Hematol. (2023) 102(10):2651–8. 10.1007/s00277-023-05374-4 37481473

[26] Sekula P Del Greco M F Pattaro C Köttgen A. Mendelian randomization as an approach to assess causality using observational data. J Am Soc Nephrol. (2016) 27(11):3253–65. 10.1681/ASN.2016010098 27486138 PMC5084898

[27] Kurki MI Karjalainen J Palta P Sipilä TP Kristiansson K Donner KM Finngen provides genetic insights from a well-phenotyped isolated population. Nature. (2023) 613(7944):508–18. 10.1038/s41586-022-05473-8 36653562 PMC9849126

[28] Jiang X Zhou R He Y Zhu T Zhang W. Causal effect of serum 25-hydroxyvitamin D levels on low back pain: a two-sample Mendelian randomization study. Front Genet. (2022) 13:1001265. 10.3389/fgene.2022.1001265 36212121 PMC9534573

[29] Chen L Guo X Hou C Tang P Zhang X Chong L The causal association between iron status and the risk of autism: a Mendelian randomization study. Front Nutr. (2022) 9:957600. 10.3389/fnut.2022.957600 36407516 PMC9669792

[30] Drury KE Schaeffer M Silverberg JI. Association between atopic disease and anemia in US children. JAMA Pediatr. (2016) 170(1):29–34. 10.1001/jamapediatrics.2015.3065 26619045

[31] Rhew K Brown JD Oh JM. Atopic disease and anemia in Korean patients: cross-sectional study with propensity score analysis. Int J Environ Res Public Health. (2020) 17(6). 10.3390/ijerph17061978 32197291 PMC7142528

[32] Wang Z He Y Cun Y Li Q Zhao Y Luo Z. Transcriptomic analysis identified SLC40A1 as a key iron metabolism-related gene in airway macrophages in childhood allergic asthma. Front Cell Dev Biol. (2023) 11:1164544. 10.3389/fcell.2023.1164544 37123407 PMC10133523

[33] Yang L Sato M Saito-Abe M Miyaji Y Shimada M Sato C Allergic disorders and risk of anemia in Japanese children: findings from the Japan environment and children’s study. Nutrients. (2022) 14(20). 10.3390/nu14204335 PMC9607270 36297019

[34] Means RT. Iron deficiency and iron deficiency anemia: implications and impact in pregnancy, fetal development, and early childhood parameters. Nutrients. (2020) 12(2):447. 10.3390/nu12020447 32053933 PMC7071168

[35] Yang W Liu B Gao R Snetselaar LG Strathearn L Bao W. Association of anemia with neurodevelopmental disorders in a nationally representative sample of US children. J Pediatr. (2021) 228:183–189.e2. 10.1016/j.jpeds.2020.09.039 33035572

[36] Park GN Kim JO Oh JW Lee S. Association between anemia and depression: the 2014, 2016, and 2018 Korea national health and nutrition examination survey. J Affect Disord. (2022) 312:86–91. 10.1016/j.jad.2022.06.015 35750091

[37] Phipps O Brookes MJ Al-Hassi HO. Iron deficiency, immunology, and colorectal cancer. Nutr Rev. (2021) 79(1):88–97. 10.1093/nutrit/nuaa040 32679587

[38] Pivina L Semenova Y Doşa MD Dauletyarova M Bjørklund G. Iron deficiency, cognitive functions, and neurobehavioral disorders in children. J Mol Neurosci. (2019) 68(1):1–10. 10.1007/s12031-019-01276-1 30778834

[39] Pichler I Del Greco MF Gögele M Lill CM Bertram L Do CB Serum iron levels and the risk of parkinson disease: a Mendelian randomization study. PLoS Med. (2013) 10(6):e1001462. 10.1371/journal.pmed.1001462 23750121 PMC3672214

[40] Ma Y Wu Y Zhang Y Jiao T Guo S Zhang D Associations between maternal complications during pregnancy and childhood asthma: a retrospective cohort study. ERJ Open Res. (2023) 9(2). 10.1183/23120541.00548-2022 PMC10086685 37057092

[41] Triche EW Lundsberg LS Wickner PG Belanger K Leaderer BP Bracken MB. Association of maternal anemia with increased wheeze and asthma in children. Ann Allergy Asthma Immunol. (2011) 106(2):131–139.e1. 10.1016/j.anai.2010.11.007 21277514 PMC3073499

[42] Shaheen SO Gissler M Devereux G Erkkola M Kinnunen TI Mcardle H Maternal iron supplementation in pregnancy and asthma in the offspring: follow-up of a randomised trial in Finland. Eur Respir J. (2020) 55(6):1902335. 10.1183/13993003.02335-2019 32139461

[43] Brigham EP McCormack MC Takemoto CM Matsui EC. Iron status is associated with asthma and lung function in US women. PLoS One. (2015) 10(2):e0117545. 10.1371/journal.pone.0117545 25689633 PMC4331366

[44] Sharma S Yang IV Schwartz DA. Epigenetic regulation of immune function in asthma. J Allergy Clin Immunol. (2022) 150(2):259–65. 10.1016/j.jaci.2022.06.002 35717251 PMC9378596

[45] Ramakrishnan K Harish PS. Hemoglobin level as a risk factor for lower respiratory tract infections. Indian J Pediatr. (2006) 73(10):881–3. 10.1007/BF02859279 17090898

[46] Winn NC Volk KM Hasty AH. Regulation of tissue iron homeostasis: the macrophage "ferrostat". JCI Insight. (2020) 5(2):e132964. 10.1172/jci.insight.132964 31996481 PMC7098718

[47] Roth-Walter F. Iron-deficiency in atopic diseases: innate immune priming by allergens and siderophores. Front Allergy. (2022) 3:859922. 10.3389/falgy.2022.859922 35769558 PMC9234869

[48] Hale LP Kant EP Greer PK Foster WM. Iron supplementation decreases severity of allergic inflammation in murine lung. PLoS One. (2012) 7(9):e45667. 10.1371/journal.pone.0045667 23029172 PMC3447873

[49] Ali MK Kim RY Brown AC Mayall JR Karim R Pinkerton JW Crucial role for lung iron level and regulation in the pathogenesis and severity of asthma. Eur Respir J. (2020) 55(4). 10.1183/13993003.01340-2019 32184317

[50] Groenman FA Rutter M Wang J Caniggia I Tibboel D Post M. Effect of chemical stabilizers of hypoxia-inducible factors on early lung development. Am J Physiol Lung Cell Mol Physiol. (2007) 293(3):L557–67. 10.1152/ajplung.00486.2006 17545484

